# Alterations in Brain Structure and Amplitude of Low-frequency after 8 weeks of Mindfulness Meditation Training in Meditation-Naïve Subjects

**DOI:** 10.1038/s41598-019-47470-4

**Published:** 2019-07-29

**Authors:** Chuan-Chih Yang, Alfonso Barrós-Loscertales, Meng Li, Daniel Pinazo, Viola Borchardt, César Ávila, Martin Walter

**Affiliations:** 1 Clinical Affective Neuroimaging Laboratory, Otto-von-Guericke University, Magdeburg, Germany; 2 Departamento de Psicología Básica, Clínica y Psicobiología, Universitat Jaume I, Castellón de la Plana, Spain; 3 Departamento de Psicología Educativa, Social y Metodología, Universitat Jaume I, Castellón de la Plana, Spain; 4 Department of Neurology, Otto-von-Guericke University, Magdeburg, Germany; 5 Department of Behavioral Neurology, Leibniz Institute for Neurobiology, Magdeburg, Germany; 6 Department of Psychiatry and Psychotherapy, Otto-von-Guericke University, Magdeburg, Germany; 7 Center of Behavioral Brain Sciences, Otto-von-Guericke University, Magdeburg, Germany; 8 Department of Psychiatry, Eberhard Karls University Tuebingen, Tuebingen, Germany

**Keywords:** Neural circuits, Translational research

## Abstract

Increasing neuroimaging evidence suggests that mindfulness meditation expertise is related to different functional and structural configurations of the default mode network (DMN), the salience network (SN) and the executive network at rest. However, longitudinal studies observing resting network plasticity effects in brains of novices who started to practice meditation are scarce and generally related to one dimension, such as structural or functional effects. The purpose of this study was to investigate structural and functional brain network changes (e.g. DMN) after 40 days of mindfulness meditation training in novices and set these in the context of potentially altered depression symptomatology and anxiety. We found overlapping structural and functional effects in precuneus, a posterior DMN region, where cortical thickness increased and low-frequency amplitudes (ALFF) decreased, while decreased ALFF in left precuneus/posterior cingulate cortex correlates with the reduction of (CES-D) depression scores. In conclusion, regional overlapping of structural and functional changes in precuneus may capture different components of the complex changes of mindfulness meditation training.

## Introduction

Mindfulness meditation has gained growing interest in psychological research. Mindfulness is defined as the process by which one attends to present-moment experiences non-judgementally, which can, for example, be developed through meditation practice^1–3^. Brain research has yielded global and local levels of differences in brain activity and connectivity related to meditation affecting several brain regions involving the default mode network (DMN), the salience network and the executive network. It is noteworthy that global changes have shown reduced connectivity between the nodes of the DMN and other networks, as well as reduced local activity and connectivity of nodes within these networks (e.g., ReHo, ALFF, nodal strength). This study aims to explore whether behavioural, structural and local functional brain changes are associated with short-term mindfulness meditation training.

Mindfulness meditation practice primarily leads to reductions in ruminative thinking, even after controlling for decrements in affective symptoms and dysfunctional beliefs^4^. In patients with affective disorders, mindfulness meditation intervention has been shown to improve anxiety and mood symptoms^5^. At a behavioural level, several studies have indicated that mindfulness-based therapy is a promising approach for treating anxiety and mood disorders^6–8^. Several previous studies have demonstrated the practice of mindfulness meditation leads to brain changes at either structural or functional levels^9–15^. To track functional changes, resting-state functional magnetic resonance imaging (rs-fMRI) has been proposed to analyse differential spontaneous modulations in the blood-oxygen-level dependent (BOLD) signal during resting conditions^16^. With rs-fMRI, researchers can characterise the brain’s spontaneous functional activities in terms of both brain network connectivity and local features, e.g. the amplitude of low-frequency fluctuations (ALFF) to characterise the amplitude of local spontaneous brain activity^17^. In contrast to the functional connectivity method, which measures the temporal synchronisation between brain areas, the ALFF method helps to localise the brain functional alterations^18^. The quantitative measurement of low-frequency oscillations, such as ALFF, provides promising tools to observe spontaneous BOLD signal alterations in regional activity. As such, it may offer insight into the direct local consequences of the previously reported structural changes. Although several previous studies have associated brain structure changes with mindfulness meditation training, plus there is evidence for a cross-sectional convergence of functional and structural changes when comparing professional meditators and novices^19^, longitudinal research into structural changes and their direct relationship would be necessary to more directly relate the changes in the two dimensions to one another and to clinical or behavioural effects, respectively.

To study brain morphometry, several methods have been proposed to investigate brain structural changes, such as voxel-based morphometry (VBM)^20^ and the cortical thickness measure (Freesurfer)^21,22^. The cortical thickness measure is preferred because it utilises brain geometry to conduct inter-subject registration, which contributes to a finer matching of homologous cortical locations than volumetric-based methods. Freesurfer is commonly applied in many cortical thickness studies^13,14,23^, and in this study, Freesurfer is used for the cortical thickness analysis given its advantages over the volume-based morphometry method. Fox *et al*. reported and meta-analysed 123 brain structural differences using several morphometric measures (e.g. cortical thickness, grey matter volume and concentration, fractional anisotropy, etc.) to show that mindfulness meditation also leads to significant brain structure changes^24^. Prior studies have pioneered longitudinal analysis on grey and white matter changes^25–27^. Tang *et al*. observed increased fractional anisotropy in the corona radiata connecting the cingulate cortex after an 11-hour integrative body-mind training (IBMT)^27^. Santarnecchi *et al*. showed a significant increase in cortical thickness in the right insula and the somatosensory cortex after an 8-weeks Mindfulness-Based Stress Reduction (MBSR) training program^25^. Pickut *et al*. reported increased grey matter density in the bilateral caudate, left cuneus, left thalamus, and left lingual gyrus in an 8-week mindfulness-based intervention randomised controlled trial (RCT) for Parkinson subjects who underwent mindfulness-based intervention^26^.

In cross-sectional studies, Kang *et al*. found increased cortical thickness in the superior frontal cortex, frontal medial prefrontal cortex, temporal areas for meditation subjects compared to the controls^13^. Finally, it has been demonstrated that the long-term practice of Sahaja Yoga Meditation leads to increased grey matter volume and regional enlargement in different cortical and subcortical brain areas which are correlated with compassion and interoceptive perception, sustained attention and self-control^28^. The recent work by Engen *et al*. investigated structural brain networks changes of long-term mental training effects on socio-affective skills^19^. These authors conducted both functional and structural analyses and found that ALFF increases in several prefrontal and insular areas during meditation relative to resting, and they also observed cortical changes.

In this longitudinal study, we investigated cortical thickness changes at the whole-brain level as well as ALFF changes at the baseline and following 40-day short-term mindfulness meditation training. The primary objective of this study was to investigate regionally overlapping longitudinal structural and functional (ALFF) changes associated with short-term mindfulness meditation training in meditation novices. We hypothesised that the repeated activation of brain regions corresponding to the brain networks recruited during meditation training might induce congruent early structural and functional activity changes within relevant circuits. Based on prior findings, these were hypothesised to concern the default mode network (DMN). We further hypothesised if these brain changes were associated with potential behavioural changes in depression and anxiety scores. The current study used the data collected in our previous work^15^.

## Results

### Demographics and behavioural measures

The primary behavioural outcome for the subjects is summarised in Table 1. The sum scores of CES-D reduced significantly (t(13) = 4.402; p < 0.001), controlled for multiple comparisons. STAI scores (trait anxiety) reduced significantly (t(13) = 2.73; p < 0.01).

**Table 1:** Primary behavioural outcome.

	TP1 (mean ± standard deviation)	TP2 (mean ± standard deviation)
CES-D	15.57 (±9.49)	8.71 (±6.06)***
STAI, state	17.93 (±4.58)	13.57 (±10.23)
STAI, trait	22.50 (±9.39)	16.36 (±9.37)**

CES-D, Center for Epidemiologic Studies Depression Scale; STAI: State-Trait Anxiety Inventory. Significant group differences: ∗∗∗significant at p < 0.001, ∗∗significant at p < 0.01.

The demographic and exploratory behavioural information for the subjects is summarised in Table 2. No significant change in the POMS sub-scores was observed. The FFMQ factor – non-reactivity to inner experience significantly increased after meditation training (t(13) = 6.2; p < 0.001, uncorrected for multiple comparisons).

**Table 2:** Subject demographic and exploratory behavioural measures. Group mean and standard deviation are listed.

	TP1 (mean ± standard deviation)	TP2 (mean ± standard deviation)
Age (years)	24.53 (±5.90)	24.64 (±5.90)
The males-to-females ratio	4/10	4/10
Minutes of meditation practice a day	0	10.84 (±8.41)
Total number of meditation days until scanning	0	39.23 (±3.63)
Total number of minutes of meditation practice until scanning	0	423.41 (±30.56)
POMS, Anger (−)	2.64 (±3.71)	3.07 (±5.90)
POMS, Fatigue (−)	4.57 (±2.77)	3.43 (±4.50)
POMS, Vigour (+)	11.85 (±5.11)	12.50 (±5.19)
POMS, Friendliness (+)	18.28 (±3.72)	19.14 (±4.27)
POMS, Tension (−)	7.64 (±3.49)	5.07 (±5.12)
POMS, Depression (−)	3.85 (±3.79)	4.21 (±7.82)
FFMQ, non-reactivity to inner experience	4.55 (±0.83)	6.10 (±1.19)***
FFMQ, observing	5.94 (±1.42)	6.48 (±1.68)
FFMQ, acting with awareness	4.12 (±1.38)	3.99 (±0.94)
FFMQ, describing	4.74 (±0.48)	4.93 (±1.41)
FFMQ, non-judgeing of experience	4.56 (±1.09)	4.92 (±0.86)

The mean values (standard deviation) for each variable are shown before (TP1) and after (TP2) the mindfulness training. POMS, Profile of Mood States; FFMQ, Five Facet Mindfulness Questionnaire. Significant group differences: ∗∗∗significant at p < 0.001, ∗∗significant at p < 0.01. (+): positive mood state factor, (−): negative mood state factor.

### Structural whole cortex analysis: cortical thickness changes following mindfulness meditation

**Figure 1:**
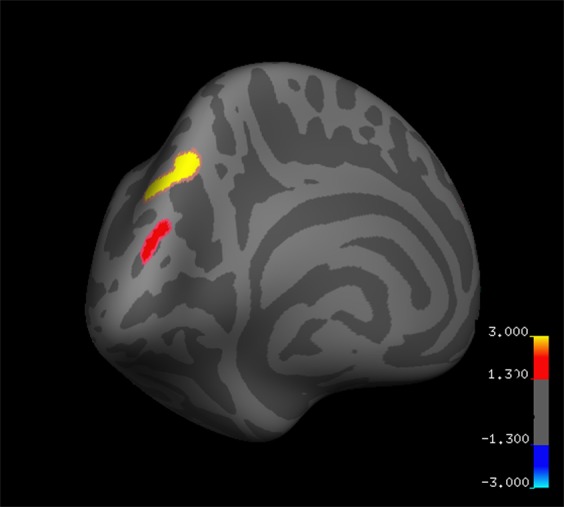
Whole brain pre-post cortical thickness changes (PC1: percent thickness change). Significant regional increases in cortical thickness following mindfulness meditation training in the left precuneus (−12.7, −72.9, 40.7; shown in yellow) and left superior parietal lobule (−8.8, −89.2, 21.4; shown in red; p < 0.05, corrected by Monte Carlo simulation). Colorbar shows the false-positive rate (-log10(p)): thresholds of −3, −1.3, 1.3, 3 correspond to p-values of −0.001, −0.05, 0.05, 0.001.

Regional cortical thickness increased significantly (PC1) in the left precuneus (MNI coordinates at peak vertex: [−12.7, −72.9, 40.7]; cluster-wise p = 0.0003) and the left superior parietal lobule (peak vertex at [−8.8, −89.2, 21.4]; cluster-wise p = 0.01641), corrected for multiple comparisons using Monte Carlo Simulation (Fig. 1).

### Whole brain ALFF analysis result: ALFF changes after mindfulness meditation

**Figure 2:**
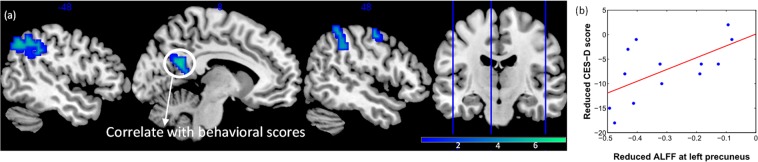
(**a**) Longitudinal ALFF decreases after mindfulness meditation training (TP1 > TP2): Left IPL (left angular gyrus), left PCC/precuneus and right IPL (right supramarginal) were found (FDR-corrected, p < 0.05). Colorbar shows the t-values. (**b**) *Post hoc* correlation of the mean ALFF at the left PCC/precuneus with CES-D. Decreased ALFF at the left PCC/precuneus was found to positively correlate with the reduction of depression score (CES-D), p = 0.024, r = 0.619.

For the functional resting state fluctuations, we found that ALFF decreased after meditation training in the left PCC/precuneus and, in addition to the structural findings, also in the bilateral IPL (p < 0.05, FDR-corrected, Fig. 2a).

### Post hoc correlation analysis results

Correlations between the whole brain percentage cortical thickness changes and ALFF with significantly changing behavioural scores (CES-D, STAI-trait) were performed.

**Figure 3:**
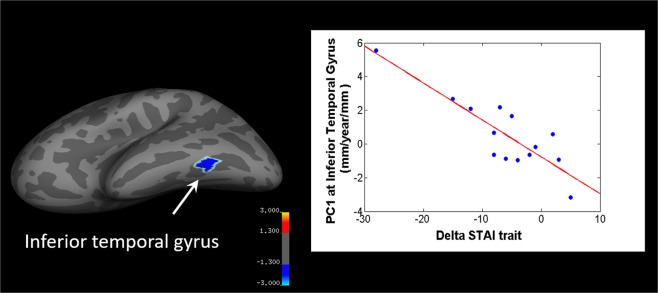
Post hoc correlations of cortical thickness changes with the delta STAI trait score. The delta STAI trait scores negatively correlate with the percent thickness change in the left inferior temporal gyrus (Monte Carlo Simulation-corrected. p < 0.05). *r* = *−0.86, p* = *0.001*. Colorbar shows the false-positive rate (−log10(p)): thresholds of −3, −1.3, 1.3, 3 correspond to p-values of −0.001, −0.05, 0.05, 0.001.

On a whole brain level, changes in STAI trait scores were negatively correlated with the percentage thickness changes in the left inferior temporal gyrus (Monte Carlo-corrected, p < 0.05, Fig. 3). For ALFF, there was no whole-brain corrected correlation of the meditation-induced changes and changes in behavioural scores.

Given the regional effects on PCC/precuneus, we extracted the delta mean ALFF values at the left PCC/precuneus and correlated these with the delta CES-D and delta STAI-trait scores, respectively. We observed a negative correlation with delta CES-D: p = 0.024, r = 0.619 (Fig. 2b). Likewise, no significant correlations with these variables were observed at the baseline ALFF. When extracting cortical thickness in precuneus, no correlation with either score was found.

## Discussion

In the present longitudinal study, we investigated both the structural and ALFF changes following 40-day short-term mindfulness meditation training and their relationship with the mood effects of the practice. The results centred on the parietal cortex with a relative increase in cortical thickness in the left precuneus, which significantly showed decreased ALFF. Furthermore, these decreases were stronger for those subjects who also showed the most reductions in depression symptoms. Additional findings concerned the upper left parietal cortex and bilateral IPL, where the cortical thickness increase in the left inferior temporal gyrus correlated with STAI reductions after 40 days.

Our findings of structural changes following the meditation training period confirmed our hypothesis about directionality based on previous results. In our study, greater thickness was observed in the left superior parietal cortex and left precuneus. A previous study demonstrated increased cortical thickness, but focuses on multiple prefrontal areas and insula regions^19^. In that study, however, the authors reported a cross-sectional increase with a long-term meditators cohort relative to meditation-naïve controls. In other cross-sectional studies, once again greater cortical thickness has been observed at the prefrontal cortex^14,29^ and in insula regions^14^ for the meditation group compared to the matched controls. The focus of the effects on the parietal cortex, as found in our study, has to be considered in contrast to other studies. In principle, one may assume two major potential sources of this difference, one related to the investigated modalities and another to the varying experimental conditions. Most of the studies performed to date have used grey matter volumetric analyses which, in contrast to our cortical thickness measures, may have biased the findings; e.g. towards subcortical areas. However, thickness changes have also been reported that have also focused on prefrontal or insula areas. Particularly the study by Santarnecchi *et al*. also applied MBSR training over 8 weeks, so the differences would have been considered minimal. Nevertheless in their study, no significant effects were found in the precuneus, but strong effects were noted in the insula and somatosensory cortices instead^25^. Furthermore, rather than using surface-based methods, those authors used voxel-based cortical thickness measures^25^. While the sources of varying structural changes in comparison to previous studies cannot be entirely identified, the current structural findings seem, however, plausible given their spatial overlap with accompanying functional changes.

Our previous study showed functional brain changes by employing resting-state functional connectivity (RSFC) and regional homogeneity (ReHo) measures after short-term mindfulness meditation training^15^. Rs-fMRI has been extensively used in recent years for investigating the neural correlates associated with mindfulness meditation training^12,15,30–33^. By employing ALFF measures in the present study, we found the decreased intensity of low-frequency oscillations in regions of the DMN (PCC/precuneus and bilateral IPL) after mindfulness training. Differential ALFF results have been observed in major depressive disorders (MDD) patients^34–36^. Increased ALFF in IPL has been found in MDD patients compared to healthy controls^36^. It is noteworthy that an rs-fMRI study by Sheline *et al*. has reported hyper-connectivity in the DMN for MDD subjects^37^.

Other than in structural studies, the functional activity and connectivity of precuneus and superior parietal cortices are more frequently reported in meditation studies. The superior parietal cortex is related to attentional function developed during the practice of mindfulness meditation^30^. In the precuneus, self-referential processing is associated with increased functional activity^38^, which decreases during mindful self-awareness^39^. Likewise, an altered RSFC of the superior parietal and precuneus has been observed in several previous meditation studies^15,40,41^. Superior parietal changes overlap with previously reported cortical thickness changes in Zen meditators^42^. Furthermore, these findings extend our prior work based on the same subjects, in which we showed that FC during meditation practice increased in the precuneus when compared to the baseline RSFC^15^. In a meta-analysis on the structural effects of meditation, the precuneus emerged as a region of structural heterogeneities in meditation practitioners^24^. Fox *et al*. suggested that the structural connectivity between the precuneus and areas of the attentional network, e.g. the superior parietal lobe, may be mediated by changes in the structural white matter at the superior longitudinal fasciculus after brief meditation training. Therefore, increments in cortical thickness in the superior parietal and precuneus after short-term meditation training may be caused by other structural changes in white matter connectivity as well as FC during the mindfulness state as a practice effect after brief mindfulness meditation.

Structural and functional changes were accompanied by reductions in depression scores and anxiety traits. To reveal an indication of the interrelation of behavioural and brain effects of meditation, we correlated the significant changes in the behavioural scores from the questionnaires (CES-D, STAI-trait) with brain changes in both dimensions. The precuneus was revealed in both structural and functional findings, and the PCC/precuneus also showed decreased ALFF, which correlated directly with the CES-D reductions. The relation between precuneus resting state behaviour and depression symptoms has been demonstrated in many studies, mostly in depressed cohorts^43,44^. Here we show that the PCC/precuneus, in its intrinsic fluctuation amplitudes, is also related to varying subclinical expressions of depressive symptomatology and their modulation by mindfulness meditation training, which has *per se* been demonstrated to act on depressive symptoms^45^. Interestingly, while we did also observe structural changes in the precuneus, they did not correlate with the amount of CES-D reductions. Furthermore, no other region correlated with this effect at the whole brain level, which places the co-occurrence of the structural and functional findings in a more complex picture. One could interpret the divergence of correlations as being indicative of stronger, more sensitive effects in the functional domain. However, it is necessary to note that for the other behavioural effect, namely reduced anxiety, we found correlation with the thickness increases in the inferior temporal gyrus.

Taken together, the longitudinal changes seemed to show a regional overlap in the precuneus, which in itself may indicate true neuronal effects observed across modalities, while their interindividual covariations with the behaviour consequences of mindfulness meditation training support the notion of a brain-wide network affected by the multitude of functional effects. Investigating this divergence and identifying the potential driving mechanisms may explain why effects on STAI reductions were found at different locations and in a distinct modality from that for the functional changes associated with CES-D. It will be necessary to perform an analysis in a larger new cohort with a control group that allows the inclusion of potential mediating variables and the control of other relevant factors, as well as potentially varying signal-to-noise ratios for our observed effects.

Along these lines, it should be noted that the findings in this study also need to be considered in their respective limitations. The small sample size (n = 14) limits the generalizability of current findings. Another limitation is that we did not recruit a control group and, therefore, these results have to be interpreted with caution. Future longitudinal RCT studies in mindfulness meditation would be of interest. Our study showed longitudinal changes in a single sample of meditation trainees across imaging modalities, which is its main contribution compared to cross-sectional studies with a control group. To establish exact effect sizes and to support generalisability, our study would have to be replicated in a larger sample, including a control condition. In this study we applied scrubbing as the head motion regressor in functional data preprocessing, and we also performed analyses without using scrubbing, which is provided in the Supplementary Materials (see the Supplementary Materials). Furthermore, the pre- and post- head motion parameters are also provided (see the Supplementary Materials).

In summary, we found that 40 days mindfulness meditation practice leads to a reduction of subclinical depression symptomatology, which is correlated with reduced resting state fluctuations in the precuneus, a region in which cortical increase following meditation has been observed in the same sample. This mechanism falls well in line with previous investigations on ALFF in depression and mindfulness meditation but needs to be placed in a more complex picture of a network-wide effect with additional structural changes in temporal regions in association with anxiety. Therefore, we conclude that functional and structural effects, despite regional overlapping, may capture different components of the complex action of mindfulness meditation training.

## Materials and Methods

The current analysis focuses on the structural and functional dataset which the functional data have been previously investigated for resting-state functional connectivity^15^. As such the reported parameters on subject intervention and data acquisition are identical, while the analysis differs in terms of the analysed modalities and respective phenomena.

### Subjects

Fourteen university students (native Spanish speakers) were enrolled to be involved in a 40-day mindfulness meditation training course. All the experiment protocols were approved by the local Ethics Committee of the Institutional Review Board of the Universitat Jaume I of Castellon (Spain). Informed consent was in accordance with the Declaration of Helsinki. The guidelines of the local Ethics Committee on research involving human participants were collected from all the participants prior to inclusion. All the subjects were screened by psychologists for the absence of any neurological or psychiatric disorder before being enrolled. Subjects reported no previous participation in meditation courses or training. All the participants except one had been reported in our previous study^15^, who was excluded due to excessive head movement during MR acquisition.

### Mindfulness meditation training

As described in our previous work^15^, the mindfulness meditation programme was structured as an 8-week training programme consisting of 45 minutes of daily home practice. The self-observation-based mindfulness meditation training programme was designed using the MBSR programme^1^, as well as acceptance and commitment therapy^46^. Training consisted of a 1.5-hour weekly session over an 8-week period. During the first hour of the session, the subjects had to perform simple physical and breathing exercises, during which Vipassana meditation was practiced by focusing their attention on thoughts without dwelling on them. Afterwards, the participants had to remain silent and witness their thoughts without centring on them. During the next sessions, the time allotted to meditation training was gradually increased without physical exercises. During the remaining half hour of the session, the participants were requested to share their experiences and were shown short videos that explained the most significant aspects of meditation and self-observation training. They were also encouraged to continue their daily meditation practice at home. The course instructor gave a meditation journal to each participant to keep track of their daily practice routine and to write down their experiences. Table 2 details the participants’ age, gender and amount of meditation practice.

### Behavioural measurements

Three self-assessment questionnaires were given to subjects at time points TP1 and TP2. The Center for Epidemiologic Studies Depression Scale (CES-D), which is a 20-item inventory^47^ was used to evaluate changes in depressive symptoms. The participants were requested to rate statements based on the previous week on a 4-point Likert scale (0–3) that scored from 0 to 60. Higher scores indicate higher levels of depression. The State-Trait Anxiety Inventory was also applied, which is a 40-item scale measuring state and trait anxiety based on a 4-point Likert scale (1–4). Scores range from 20 to 80, and high scores relate to high levels of anxiety^48^. This portion of behavioural measurement is from part of our previous work^15^.

To explore the dimensions of potential effects on mindfulness, we further accessed the mindfulness measure Five Facet Mindfulness Questionnaire (FFMQ) before and after the meditation training^49^. Specifically, we assessed the facets of non-reactivity to inner experience, observation, acting with awareness, describing and non-judgement of experience. The participants responded on a 9-point Likert-type scale (1 = never or rarely, 9 = almost always or always true) both before (TP1) and after (TP2) meditation training. Higher scores indicates better mindfulness skills. We also used the Profile of Mood States (POMS), specifically the abbreviated Spanish version^50^ of the original POMS^51^. It is a 44-item inventory that measures current mood state by rating statements on a 5-point Likert scale (0 = not at all, 4 = extremely) and consists of six subscales: anger, fatigue, tension, depression, vigour and friendliness.

### MR sequence parameters

The MR measurements were performed on a 1.5 T Siemens AVANTO scanner (Siemens Erlangen, Germany). The current study used previous data^15^. Structural images were acquired with a magnetisation-prepared rapid gradient-echo (MP-RAGE) sequence (TR = 2200 ms, TE = 3.79 ms, flip angle (FA) = 15°, 160 slices, matrix size = 256 × 256, field-of-view (FOV) = 256 mm × 256 mm, and slice thickness = 1 mm). For resting-state fMRI acquisition, a standard EPI sequence was used (TR = 2300 ms, TE = 55 ms, FA = 90°, FOV = 224 mm × 224 mm, matrix size = 64 × 64, and slice thickness = 4 mm) with 25 axial slices for whole brain coverage. Finally, an extra gradient field mapping sequence (GRE field mapping) was acquired, followed by another EPI sequence (TR = 487 ms, TE1 = 8 ms, TE2 = 12.76 ms, FA = 65°, FOV = 224 mm × 224 mm, matrix size = 64 × 64, and slice thickness = 4 mm) with 25 slices with the same coverage used in the first EPI sequence. GRE field mapping sequence was acquired to correct for field-inhomogeneity artefacts in EPI.

### Structural data pre-processing pipeline

Cortical thickness was calculated using FreeSurfer (http://surfer.nmr.mgh.harvard.edu), version 5.3. T1 MPRAGE images were utilised in FreeSurfer to compute a 3-dimensional model of cortical surface reconstructions. The technical details of the processing procedure are described in previous publications^21,22,52–54^.

In short, non-brain tissue removal, automated Talairach transformation, volumetric structures segmentation^21,54^, cortical surface inflation to an average spherical surface, intensity normalisation, and automated topology correction were executed^55,56^. Surface deformation was used to detect the most significant intensity shift to calculate the boundary between grey/white matter and the pial surface. GM/WM/CSF segmentation quality was visually examined in each subject, and the subjects with inaccurate segmentation were then excluded. Each hemisphere was parcellated automatically into 74 distinct cortical areas, and the thickness and volume of these brain regions were calculated^57^.

#### Longitudinal structural data processing: cortical thickness – longitudinal two-stage model

Vertex-wise analyses of cortical thickness changes were performed with Freesurfer. Firstly, each subject’s image data were smoothed by using a Gaussian kernel with an FWHM of 15 mm. To investigate the changes in cortical thickness associated with mindfulness meditation training, the *longitudinalTwoStageModel* implemented in FreeSurfer was executed (https://surfer.nmr.mgh.harvard.edu/fswiki/LongitudinalTwoStageModel^59^). The first stage reduces the temporal data within each subject to a single statistic (percent change). The second stage correlates it with a behavioural covariate^59^ (see the *Post hoc Correlation Analysis*).

An inverse consistent registration was utilised to generate an unbiased within-subject template and images^58,60^. The reliability and statistical power are significantly increased by using the common information from the within-subject template to initialise the processing steps (skull stripping, Talairach transforms, atlas registration, spherical surface maps and parcellations)^59^.

The quality assurance toolbox (https://surfer.nmr.mgh.harvard.edu/fswiki/QATools) in FreeSurfer was used to test the quality of the surface reconstruction and segmentation. In order to calculate the cortical thickness changes after mindfulness training, the percent change (pc1) was used to obtain a cortical measure. PC1 is the rate of change (*(thickness2 - thickness1)/(time2 - time1)*, unit: mm/year) in relation to the thickness at the first time point:

$$pc1=\frac{\frac{(Thickness\,at\,TP2-Thickness\,at\,TP1)}{TP2-TP1}}{Thickness\,at\,TP1}(unit:\frac{mm}{year}/mm)$$

It describes the percentage thickening/thinning at a given cortical location.

### Rs-fMRI data preprocessing

B0 inhomogeneity correction was performed to reduce static field inhomogeneity using an FSL *EpiUnwarping* tool (http://surfer.nmr.mgh.harvard.edu/fswiki/epidewarp.fsl)^61^. The similar rs-fmri data preprocessing procedure was carried out as in our previous publication^15^. The first 10 images for each subject were discarded to allow for steady-state longitudinal magnetisation. The remaining images were then preprocessed by Statistical Parametric Mapping (SPM8, http://www.fil.ion.ucl.ac.uk/spm/) and Data Processing Assistant for the resting state fMRI (DPARSF Version 4.3^62^). Functional images were slice-time corrected. Motion correction was performed by using a least squares approach and a six-parameter (rigid body) linear transformation. One subject was excluded according to the criterion that head motion was restricted to less than 2 mm of displacement or 2 degrees of rotation in any direction. The frame-wise displacement value (FD_Power) showed no significant difference between the time points (t = 1.488, p=0.162; see Supplementary Table 1). Spatial normalisation to MNI space was carried out by using the unified segmentation of the T1-weighted acquired images. The extracted normalisation parameters from segmentation were applied to normalise the functional volumes for each participant (normalised images were then resampled to 3 mm isotropic voxels). Nuisance variables were regressed out (head motion parameters, white matter signal, cerebrospinal fluid signal). Scrubbing with regression strategy was used to reduce the motion effect from the subjects^63^. The linear trends of the BOLD signals were regressed.

#### ALFF analysis

We calculated ALFF by using DPARSF v 4.3. The time series for each voxel was first band-pass filtered (0.01–0.1 Hz) and then fast Fourier-transformed (FFT) to acquire a power spectrum in the frequency domain. The square root of the power spectrum was obtained and averaged across a frequency of 0.01–0.1 Hz at each voxel. The averaged square root was then known as ALFF. Furthermore, the ALFF of each voxel for each participant was divided by the global mean ALFF for standardisation^17^. Finally, the whole-brain-mean scaled ALFF maps were smoothed by applying a 6-mm full-width-at-half-maximum (FWHM) Gaussian kernel prior to the statistical analysis.

### Statistical analysis

#### Behavioural data analysis

Changes in the Depression (CES-D) and Anxiety scores (STAI-state and STAI-trait) were assessed by paired-t tests with significance accepted for Bonferroni corrected at p < 0.05 for three tests, based on prior publications on antidepressant effects^45^. To further explore the dimensions of behavioural effects, the mindfulness questionnaires (FFMQ) and Profiles of Mood States (POMS) questionnaires were assessed for any relevant changes in the respective subscales with no prior hypothesis on an individual item. The results are considered significant for an uncorrected p < 0.05 at an exploratory level.

#### Structural data analysis

One-sample t-tests were performed by using the percent thickness change as the point measure. To correct for multiple comparisons, the Monte-Carlo simulation (*mc-z*; synthesised, smoothed z-field) within FreeSurfer was used to correct for multiple comparisons. The results were then smoothed by the residual and repeated for 10000 iterations with a threshold of *p* < 0.05 (two-tailed).

#### ALFF analysis

A voxel-wise ALFF analysis was performed to assess the whole brain amplitude of low-frequency fluctuation changes in mindfulness meditation training. A paired t-test was performed to test the whole brain longitudinal ALFF differences (two-tailed). Multiple comparisons were corrected at an FDR cluster level of p < 0.05 with a conservative initial voxel height threshold of p < 0.001.

#### Post hoc correlation analysis

Regression models (one-sample t-tests) included the percent thickness change as an independent factor and the above behavioural scores changes as dependent factors. Age and gender were included as nuisance covariates in all the GLM analyses. Respective multiple comparisons correction was carried out to correct type I errors.

A *post hoc* Pearson correlation analysis was also calculated to investigate the relationship between the ALFF values and behavioural scores (a statistical significance level of p < 0.05 was used). The analysis was done only on those items from the questionnaires that revealed a significant main effect of intervention. Based on our behavioural results in this study, the cortical thickness and ALFF changes were correlated with the significant changes in behavioural scores from questionnaires CES-D and STAI-trait.

## Supplementary information

Supplementary Materials

## Data Availability

The datasets are available from the corresponding author.

## References

[1] Kabat-Zinn, J. & Clinic, U. of M. M. C. S. R. *Full Catastrophe Living: Using the Wisdom of Your Body and Mind to Face Stress, Pain, and Illness*. (Delta Trade Paperbacks, 1990).

[2] Pagnini F, Philips D (2015). Being mindful about mindfulness. The Lancet Psychiatry.

[3] Slagter, H. A., Davidson, R. J. & Lutz, A. Mental Training as a Tool in the Neuroscientific Study of Brain and Cognitive Plasticity. *Front. Hum. Neurosci*. **5** (2011). 10.3389/fnhum.2011.00017 PMC3039118 21347275

[4] Ramel, W., Goldin, P. R., Carmona, P. E. & McQuaid, J. R. The effects of mindfulness meditation on cognitive processes and affect in patients with past depression. *Cognit. Ther. Res*., 10.1023/B:COTR.0000045557.15923.96 (2004).

[5] Hofmann Stefan G., Sawyer Alice T., Witt Ashley A., Oh Diana (2010). The effect of mindfulness-based therapy on anxiety and depression: A meta-analytic review. Journal of Consulting and Clinical Psychology.

[6] Goyal M (2014). Meditation programs for psychological stress and well-being: a systematic review and meta-analysis. JAMA Intern Med.

[7] Marchand WR (2014). Neural mechanisms of mindfulness and meditation: Evidence from neuroimaging studies. World J. Radiol..

[8] Mars Thomas S., Abbey Hilary (2010). Mindfulness meditation practise as a healthcare intervention: A systematic review. International Journal of Osteopathic Medicine.

[9] Davanger S, Ellingsen O, Holen A, Hugdahl K (2010). Meditation-specific prefrontal cortical activation during acem meditation: an fMRI study. Percept. Mot. Skills.

[10] Farb NAS, Segal ZV, Anderson AK (2013). Mindfulness meditation training alters cortical representations of interoceptive attention. Soc. Cogn. Affect. Neurosci..

[11] Hölzel Britta K., Carmody James, Vangel Mark, Congleton Christina, Yerramsetti Sita M., Gard Tim, Lazar Sara W. (2011). Mindfulness practice leads to increases in regional brain gray matter density. Psychiatry Research: Neuroimaging.

[12] Jang JH (2011). Increased default mode network connectivity associated with meditation. Neurosci. Lett..

[13] Kang DH (2013). The effect of meditation on brain structure: Cortical thickness mapping and diffusion tensor imaging. Soc. Cogn. Affect. Neurosci..

[14] Lazar SW (2005). Meditation experience is associated with increased cortical thickness. Neuroreport.

[15] Yang C (2016). State and Training Effects of Mindfulness Meditation on Brain Networks Reflect Neuronal Mechanisms of Its Antidepressant Effect. Neural Plast..

[16] Fox MD, Raichle ME (2007). Spontaneous fluctuations in brain activity observed with functional magnetic resonance imaging. Nature Reviews Neuroscience.

[17] Zang YF (2007). Altered baseline brain activity in children with ADHD revealed by resting-state functional MRI. Brain Dev..

[18] Lu H., Zuo Y., Gu H., Waltz J. A., Zhan W., Scholl C. A., Rea W., Yang Y., Stein E. A. (2007). Synchronized delta oscillations correlate with the resting-state functional MRI signal. Proceedings of the National Academy of Sciences.

[19] Engen, H. G., Bernhardt, B. C., Skottnik, L., Ricard, M. & Singer, T. Structural changes in socio-affective networks: Multi-modal MRI findings in long-term meditation practitioners. *Neuropsychologia*, 10.1016/j.neuropsychologia.2017.08.024 (2017). 10.1016/j.neuropsychologia.2017.08.024 28842274

[20] Ashburner J, Friston KJ (2000). Voxel-Based Morphometry—The Methods. Neuroimage.

[21] Fischl B (2004). Automatically Parcellating the Human Cerebral Cortex. Cereb. Cortex.

[22] Fischl B, Dale AM (2000). Measuring the thickness of the human cerebral cortex from magnetic resonance images. Proc. Natl. Acad. Sci..

[23] Yang H (2016). Neurochemical and neuroanatomical plasticity following memory training and yoga interventions in older adults with mild cognitive impairment. Front. Aging Neurosci..

[24] Fox KCR (2014). Is meditation associated with altered brain structure? A systematic review and meta-analysis of morphometric neuroimaging in meditation practitioners. Neuroscience and Biobehavioral Reviews.

[25] Santarnecchi E (2014). Interaction between neuroanatomical and psychological changes after mindfulness-based training. PLoS One.

[26] Pickut BA (2013). Mindfulness based intervention in Parkinson’s disease leads to structural brain changes on MRI: A randomized controlled longitudinal trial. Clin. Neurol. Neurosurg..

[27] Tang Y (2010). Short-term meditation induces white matter changes in the anterior cingulate..

[28] Hernández S, Suero J, Barros A, Rubia K (2016). Increased grey matter associated with long-Term Sahaja yoga meditation: A voxel-based morphometry study. PLoS One.

[29] Afonso, R. F. *et al*. Greater cortical thickness in elderly female yoga practitioners-A cross-sectional study. *Front. Aging Neurosci*., 10.3389/fnagi.2017.00201 (2017). 10.3389/fnagi.2017.00201 PMC5476728 28676757

[30] Creswell JD (2016). Alterations in resting-state functional connectivity link mindfulness meditation with reduced interleukin-6: A randomized controlled trial. Biol. Psychiatry.

[31] Froeliger Brett, Garland Eric L., Kozink Rachel V., Modlin Leslie A., Chen Nan-Kuei, McClernon F. Joseph, Greeson Jeffrey M., Sobin Paul (2012). Meditation-State Functional Connectivity (msFC): Strengthening of the Dorsal Attention Network and Beyond. Evidence-Based Complementary and Alternative Medicine.

[32] Hasenkamp, W. & Barsalou, L. W. Effects of Meditation Experience on Functional Connectivity of Distributed Brain Networks. *Front. Hum. Neurosci*. **6** (2012). 10.3389/fnhum.2012.00038 PMC3290768 22403536

[33] Taylor Véronique A., Daneault Véronique, Grant Joshua, Scavone Geneviève, Breton Estelle, Roffe-Vidal Sébastien, Courtemanche Jérôme, Lavarenne Anaïs S., Marrelec Guillaume, Benali Habib, Beauregard Mario (2012). Impact of meditation training on the default mode network during a restful state. Social Cognitive and Affective Neuroscience.

[34] Chen VC-H (2017). Assessment of brain functional connectome alternations and correlation with depression and anxiety in major depressive disorders. PeerJ.

[35] Wang L (2012). Amplitude of Low-Frequency Oscillations in First-Episode, Treatment-Naive Patients with Major Depressive Disorder: A Resting-State Functional MRI Study. PLoS One.

[36] Xu Z (2018). Altered resting-state brain activities in drug-naïve major depressive disorder assessed by fMRI: Associations with somatic symptoms defined by Yin-Yang theory of the traditional Chinese Medicine. Front. Psychiatry.

[37] Sheline Y. I., Price J. L., Yan Z., Mintun M. A. (2010). Resting-state functional MRI in depression unmasks increased connectivity between networks via the dorsal nexus. Proceedings of the National Academy of Sciences.

[38] Herwig U, Kaffenberger T, Jäncke L, Brühl AB (2010). Self-related awareness and emotion regulation. Neuroimage.

[39] Lutz J (2016). Altered processing of self-related emotional stimuli in mindfulness meditators. Neuroimage.

[40] Garrison KA, Scheinost D, Constable RT, Brewer JA (2014). BOLD signal and functional connectivity associated with loving kindness meditation. Brain Behav..

[41] Shao R, Keuper K, Geng X, Lee TMC (2016). Pons to Posterior Cingulate Functional Projections Predict Affective Processing Changes in the Elderly Following Eight Weeks of Meditation Training. EBioMedicine.

[42] Grant JA, Courtemanche J, Duerden EG, Duncan GH, Rainville P (2010). Cortical Thickness and Pain Sensitivity in Zen Meditators. Emotion.

[43] Li Gaizhi, Rossbach Kathryn, Zhang Aixia, Liu Penghong, Zhang Kerang (2018). Resting-state functional changes in the precuneus within first-episode drug-naive patients with MDD. Neuropsychiatric Disease and Treatment.

[44] Peng Daihui, Liddle Elizabeth B., Iwabuchi Sarina J., Zhang Chen, Wu Zhiguo, Liu Jun, Jiang Kaida, Xu Lin, Liddle Peter F, Palaniyappan Lena, Fang Yiru (2015). Dissociated large-scale functional connectivity networks of the precuneus in medication-naïve first-episode depression. Psychiatry Research: Neuroimaging.

[45] MARCHAND WILLIAM R. (2012). Mindfulness-Based Stress Reduction, Mindfulness-Based Cognitive Therapy, and Zen Meditation for Depression, Anxiety, Pain, and Psychological Distress. Journal of Psychiatric Practice.

[46] Hayes SC (2006). Acceptance and commitment therapy: model, processes and outcomes. Behav. Res. Ther..

[47] Radloff LS (1977). The CES-D Scale: A Self-Report Depression Scale for Research in the General Population. Appl. Psychol. Meas..

[48] Spielberger, C. D., Gorsuch, R. L., Lushene, P. R., Vagg, P. R. & Jacobs, A. G. *Manual for the State-Trait Anxiety Inventory (Form Y)*. *Manual for the statetrait anxiety inventory STAI*, 10.1007/978-1-4419-9893-4 (1983).

[49] Baer RA, Smith GT, Hopkins J, Krietemeyer J, Toney L (2006). Using Self-Report Assessment Methods to Explore Facets of Mindfulness. Assessment.

[50] Andrade E (2010). Factor structure and invariance of the POMS Mood State Questionnaire in Spanish. Span. J. Psychol..

[51] Shahid Azmeh, Wilkinson Kate, Marcu Shai, Shapiro Colin M. (2011). Profile of Mood States (POMS). STOP, THAT and One Hundred Other Sleep Scales.

[52] Dale AM, Fischl B, Sereno MI (1999). Cortical surface-based analysis: I. Segmentation and surface reconstruction. Neuroimage.

[53] Desikan RS (2006). An automated labeling system for subdividing the human cerebral cortex on MRI scans into gyral based regions of interest. Neuroimage.

[54] Fischl B (2002). Whole brain segmentation: Automated labeling of neuroanatomical structures in the human brain. Neuron.

[55] Fischl B, Liu A, Dale AM (2001). Automated manifold surgery: Constructing geometrically accurate and topologically correct models of the human cerebral cortex. IEEE Trans. Med. Imaging.

[56] Ségonne F, Pacheco J, Fischl B (2007). Geometrically accurate topology-correction of cortical surfaces using nonseparating loops. IEEE Trans. Med. Imaging.

[57] Destrieux C, Fischl B, Dale A, Halgren E (2010). Automatic parcellation of human cortical gyri and sulci using standard anatomical nomenclature. Neuroimage.

[58] Reuter Martin, Rosas H. Diana, Fischl Bruce (2010). Highly accurate inverse consistent registration: A robust approach. NeuroImage.

[59] Reuter Martin, Schmansky Nicholas J., Rosas H. Diana, Fischl Bruce (2012). Within-subject template estimation for unbiased longitudinal image analysis. NeuroImage.

[60] Reuter Martin, Fischl Bruce (2011). Avoiding asymmetry-induced bias in longitudinal image processing. NeuroImage.

[61] Smith, S. M. *et al*. Advances in functional and structural MR image analysis and implementation as FSL. In *NeuroImage* **23** (2004). 10.1016/j.neuroimage.2004.07.051 15501092

[62] Yan CG, Wang XD, Zuo XN, Zang YF (2016). DPABI: Data Processing & Analysis for (Resting-State) Brain Imaging. Neuroinformatics.

[63] Yan C (2013). A comprehensive assessment of regional variation in the impact of head micromovements on functional connectomics. Neuroimage.

