# Supplementary material for: Alterations in Brain Structure and Amplitude of Low-frequency after 8 weeks of Mindfulness Meditation Training in Meditation-Naïve Subjects
Source: Sci Rep. 2019 Jul 29;9:10977. doi: 10.1038/s41598-019-47470-4 (PMC6662752; doi:10.1038/s41598-019-47470-4)
Supplement: Supplementary file 1 — Supplementary Materials [file 41598_2019_47470_MOESM1_ESM.pdf]

TITLE: Structural and Amplitude of Low-frequency Alterations after 8 weeks of Mindfulness Meditation Training in Meditation-Naïve Subjects.

Chuan-Chih Yang[1], Alfonso Barrós-Loscertales[*2], Meng Li[1,4], Daniel Pinazo[3], Viola Borchardt[1], César Ávila[2], Martin Walter[1,5,6,7,8]

[1] Clinical Affective Neuroimaging Laboratory, Otto-von-Guericke University, Magdeburg, Germany.

[2] Departamento de Psicología Básica, Clínica y Psicobiología, Universitat Jaume I, Castellón de la Plana, Spain.

[3] Departamento de Psicología Educativa, Social y Metodología, Universitat Jaume I, Castellón de la Plana, Spain.

[4] Department of Neurology, Otto-von-Guericke University, Magdeburg, Germany.

[5] Department of Behavioral Neurology, Leibniz Institute for Neurobiology, Magdeburg, Germany.

[6] Department of Psychiatry and Psychotherapy, Otto-von-Guericke University, Magdeburg, Germany.

[7] Center of Behavioral Brain Sciences, Otto-von-Guericke University, Magdeburg, Germany.

[8] Department of Psychiatry, Eberhard Karls University Tuebingen, Tuebingen, Germany.

*Corresponding author: barros@uji.es

Supplementary Materials

ALFF analysis (with no scrubbing)

We calculated ALFF by using DPARSF v 4.3. The time series for each voxel was fast Fourier-transformed (FFT) to acquire a power spectrum in the frequency domain. The square root of the power spectrum was obtained and averaged across a frequency of 0.01–0.1 Hz at each voxel. The averaged square root was thus known as ALFF. Further, the ALFF of each voxel for each participant was divided by the global mean ALFF for standardisation. Finally, the whole-brain-mean scaled ALFF maps were smoothed by applying a 6-mm full-width-at-half-maximum (FWHM) Gaussian kernel prior to the statistical analysis. Scrubbing was excluded as the deletion of time points breaks the temporal continuity of data and is not suitable for ALFF analysis.

Statistical Analysis

The voxel-wise ALFF analysis was performed to assess the whole brain amplitude of low-frequency fluctuations changes in mindfulness meditation training. A paired t-test was performed to test the whole brain longitudinal ALFF differences (two-tailed). Multiple comparisons were corrected at the FWE cluster level of $p<0.05$ with a conservative initial voxel height threshold of $p<0.001$.

A *post hoc* Pearson correlation analysis was also calculated to investigate the relation between the ALFF values and behavioural scores. As these analyses were exploratory in nature, a statistical significance level of $p<0.05$ was used. The analysis was used with only those items from the questionnaires that revealed a significant main effect of intervention. Based on our behavioural results herein, the ALFF changes were correlated with the significant changes in the behavioural scores from questionnaires CES-D and STAI-trait.

The whole brain ALFF analysis result: ALFF changes after mindfulness meditation

We found that ALFF decreased after meditation training at the left IPL, PCC/precuneus, middle temporal gyrus and MPFC ($p<0.05$, FWE-corrected, **Supplementary Figure 1**).

Post hoc correlation analysis results

Correlations between the whole brain ALFF with significantly changing behavioural scores (CES-D, STAI-trait) were performed. There was no whole-brain corrected correlation of the meditation-induced changes and changes in the behavioural scores.

Given the regional effects on PCC/precuneus, we extracted the delta mean ALFF values at PCC/precuneus and correlated these with the delta CES-D and delta STAI-trait scores, respectively. No correlations of behavioural scores were observed. Likewise, no significant correlations with these variables were found at the baseline ALFF.

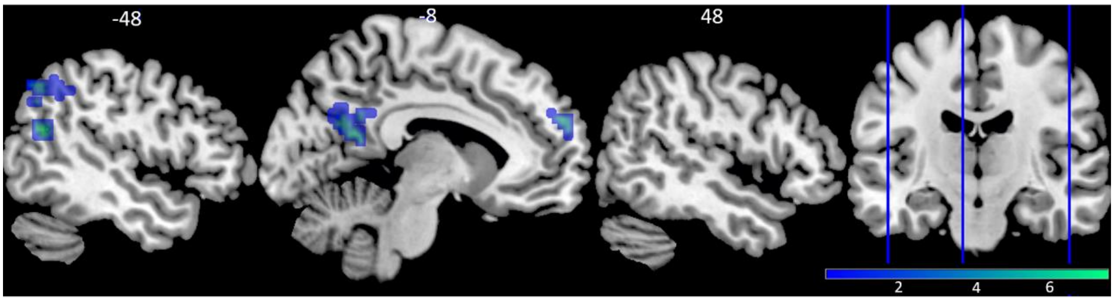

Supplementary Figure 1. Longitudinal ALFF decreased after mindfulness meditation training (TP1 Rest > TP2 Rest)*: Left IPL (left angular gyrus, -42,-60,45;t=5.97),* PCC/*precuneus (6,-63,27;t=8.57), middle temporal gyrus (-45,-60,21;t=9.34) and MPFC (-9,57,27;t=5.75) were found. No correlations of behavioural scores were observed.*

In Supplementary Table 2, the pre- and post-head motion parameters are provided, and the translation and rotation information and the FD values are listed. The frame-wise displacement value (FD_Power) showed no significant difference between the time points (t=1.488, p=0.162), as seen in Supplementary Table 1.

Supplementary Table 1.

Paired Samples Statistics

		Mean	N	Std. Deviation	Std. Error Mean
Pair 1	mean_FD_power_TP1	.1828	13	.05191	.01440
	mean_FD_power_TP2	.1572	13	.05205	.01444

Paired Samples Test

		Paired Differences					t	df	Sig. (2-tailed)
		Mean	Std. Deviation	Std. Error Mean	95% Confidence Interval of the Difference				
					Lower	Upper			
Pair 1	mean_FD_power_TP1 - mean_FD_power_TP2	.02569	.06224	.01726	-.01192	.06330	1.488	12	.162

Supplementary Table 2.

TP 1	Subject ID	mean(abs(Tx))	mean(abs(Ty))	mean(abs(Tz))	mean(abs(Rx))	mean(abs(Ry))	mean(abs(Rz))	mean RMS	mean relative RMS (mean FD_VanDijk)	mean FD_Power	Number of FD_Power>0.5	Percent of FD_Power>0.5
	sub3	0.0151983	0.07273802	0.1809405	0.04480527	0.03665587	0.04357895	0.1986725	0.02547175	0.1155779	4	0.01793722
	sub5	0.01409969	0.06544304	0.04835991	0.05569284	0.08225464	0.05139342	0.09030822	0.03202484	0.1419358	2	0.00896861
	sub7	0.02236215	0.05784394	0.1290654	0.05501336	0.04318846	0.05071814	0.1500372	0.0405811	0.1847159	6	0.02690583
	sub8	0.02167207	0.1079612	0.2114903	0.06641303	0.04147747	0.06100735	0.2418854	0.04291214	0.1639321	9	0.04035874
	sub12	0.02839788	0.05476167	0.0802863	0.0405415	0.05048683	0.05231052	0.109452	0.04781977	0.2136102	4	0.01793722
	sub14	0.01971385	0.08628262	0.1160828	0.1054857	0.02689664	0.08749514	0.1515958	0.03710793	0.1744266	12	0.05381166
	sub15	0.01628963	0.09379231	0.09320236	0.0902286	0.03574942	0.03838046	0.1414177	0.0324727	0.1750566	16	0.07174888
	sub17	0.02571213	0.05907959	0.1176643	0.113068	0.03520642	0.03856588	0.1458289	0.04308352	0.1945846	6	0.02690583
	sub18	0.02022622	0.04874388	0.06141207	0.06093529	0.08205073	0.05779998	0.09055626	0.02569603	0.1917711	8	0.03587444
	sub21	0.03046562	0.1378536	0.1993605	0.0627382	0.05788174	0.04845473	0.2486094	0.03964463	0.1685577	6	0.02690583
	sub22	0.03234156	0.06289352	0.1092133	0.09995231	0.03702078	0.1132825	0.1406438	0.07541092	0.3299748	38	0.1704036
	sub23	0.01532091	0.05783718	0.03818827	0.04978103	0.05421904	0.02229377	0.07629214	0.05734548	0.1906727	9	0.04035874
	sub26	0.02232888	0.07088732	0.1590653	0.05606171	0.04331838	0.0289808	0.1799266	0.03573045	0.1321826	3	0.01345291

TP 2	Subject ID	mean(abs(Tx))	mean(abs(Ty))	mean(abs(Tz))	mean(abs(Rx))	mean(abs(Ry))	mean(abs(Rz))	mean RMS	mean relative RMS (mean FD_VanDijk)	mean FD_Power	Number of FD_Power>0.5	Percent of FD_Power>0.5
	sub3	0.01539552	0.03478931	0.05138207	0.05197914	0.06700348	0.03541032	0.06742325	0.01344869	0.0728512	0	0
	sub5	0.01812462	0.05690424	0.05060574	0.08261732	0.03934961	0.03626015	0.08765652	0.04128302	0.218802	21	0.0941704
	sub7	0.03078545	0.07007352	0.1293621	0.1768892	0.0867019	0.04845887	0.1560144	0.02070438	0.1166197	1	0.004484305
	sub8	0.01582044	0.04621254	0.06078308	0.02740242	0.04297189	0.04497996	0.08219848	0.02044382	0.1030081	1	0.004484305
	sub12	0.0253662	0.07370546	0.102986	0.07386375	0.05692132	0.05133243	0.1393431	0.04655124	0.2294676	7	0.03139013
	sub14	0.01699523	0.1095702	0.1047364	0.05103749	0.03535276	0.03425994	0.1581724	0.03756434	0.1586316	12	0.05381166
	sub15	0.01700554	0.05077497	0.08818791	0.06414983	0.03636336	0.03996101	0.1103746	0.03849127	0.2069613	22	0.09865471
	sub17	0.03174098	0.09882186	0.1554137	0.09652759	0.08153439	0.07893983	0.1922013	0.01974875	0.1100464	0	0
	sub18	0.02509003	0.0967653	0.06109133	0.07746879	0.04166638	0.03899126	0.1290893	0.05473764	0.2175229	8	0.03587444
	sub21	0.03444302	0.02587306	0.0554224	0.05893781	0.04865121	0.04134564	0.07899936	0.03178182	0.1711262	7	0.03139013
	sub22	0.01497928	0.07248993	0.1357867	0.06164489	0.04233907	0.03608474	0.1586662	0.04329636	0.1611382	7	0.03139013
	sub23	0.009044006	0.0530499	0.03526105	0.07179832	0.02623241	0.019063	0.07265462	0.0416927	0.1740319	3	0.01345291
	sub26	0.026864	0.02434812	0.1300451	0.04846036	0.01791987	0.05473478	0.1379966	0.02631477	0.1027692	3	0.01345291
